# Supplementary material for: Identification of Novel HBV/HDV Entry Inhibitors by Pharmacophore- and QSAR-Guided Virtual Screening
Source: Viruses. 2021 Jul 29;13(8):1489. doi: 10.3390/v13081489 (PMC8402622; doi:10.3390/v13081489)
Supplement: Supplementary file 1 [file viruses-13-01489-s001.zip › viruses-1291846-supplementary.pdf]

Identification of novel HBV/HDV entry inhibitors by pharmacophore- and QSAR-guided virtual screening

Michael Kirstgen[1], Simon Franz Müller[1], Kira Alessandra Alicia Theresa Lowjaga[1], Nora Goldmann[2], Felix Lehmann[2], Sami Alakurtti[3,4], Jari Yli-Kauhaluoma[3], Karl-Heinz Baringhaus[5], Reimar Krieg[6], Dieter Glebe[2,7] and Joachim Geyer[1*]

1 Institute of Pharmacology and Toxicology, Faculty of Veterinary Medicine, Justus Liebig University Giessen, 35392 Giessen, Germany
2 Institute of Medical Virology, National Reference Center for Hepatitis B Viruses and Hepatitis D Viruses, Justus Liebig University Giessen, 35392 Giessen, Germany
3 Drug Research Program, Division of Pharmaceutical Chemistry and Technology, Faculty of Pharmacy, University of Helsinki, Viikinkaari 5 E (P.O. Box 56), FI-00014 Helsinki, Finland
4 VTT Technical Research Centre of Finland, Biologinkuja 7, (P.O. Box 1000) FI-02044 Espoo, Finland
5 Sanofi-Aventis Deutschland GmbH, 65926 Frankfurt, Germany
6 Institute of Anatomy II, University Hospital Jena, Teichgraben 7, 07743 Jena, Germany
7 German Center for Infection Research (DZIF), Partner site Giessen-Marburg-Langen, 35392 Giessen, Germany
* Correspondence: Prof. Dr. Joachim Geyer, Institute of Pharmacology and Toxicology, Biomedical Research Center Seltersberg (BFS), Schubertstr. 81, 35392 Giessen, Germany
Phone: +49 641 99 38404
FAX: +49 641 99 38409
E-Mail: Joachim.M.Geyer@vetmed.uni-giessen.de

CONTENT:

Supplementary Figure S1
Supplementary Figure S2
Supplementary Figure S3

Supplementary Figure

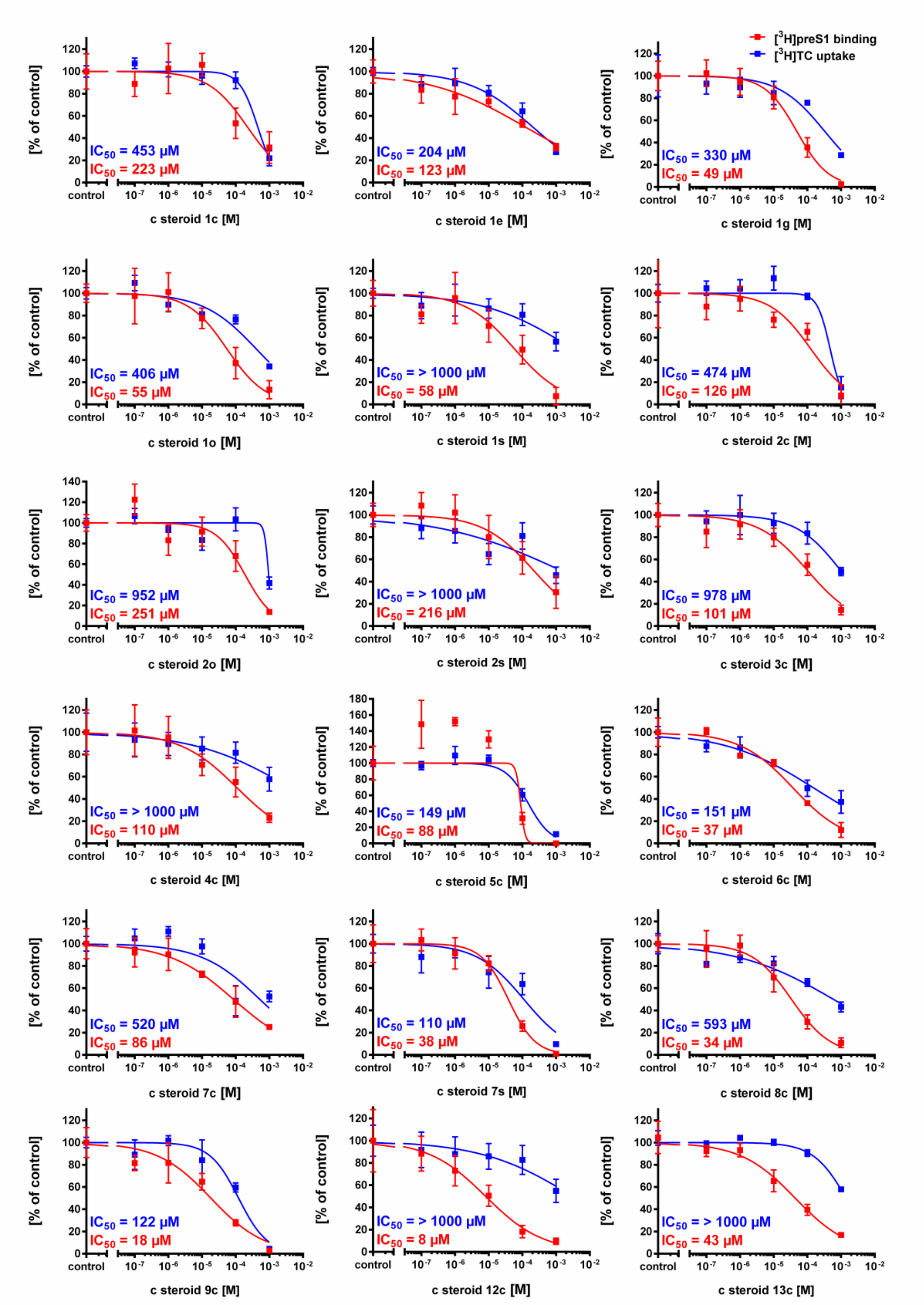

Supplementary Figure S1. Determination of IC_{50} values of 18 arylmethylamino steroids for inhibition of [^{3}H]preS1 peptide binding to NTCP and [^{3}H]TC transport via NTCP at increasing inhibitor concentrations (0.1 – 1,000 µM). NTCP-HEK293 cells were seeded onto 96-well plates and were

incubated with tetracycline to induce expression of NTCP. Cells without tetracycline treatment were used as 0% controls for both assays. Bile acid transport experiments were performed with 1 µM [^{3}H]TC and binding experiments were performed with 5 nM [^{3}H]preS1-peptide. Both assays were performed over 10 min at 37 °C with increasing concentrations of the indicated inhibitors. Control experiments were performed with solvent alone (set to 100%). The mean of the 0% control was subtracted to calculate net [^{3}H]TC transport rates (shown in blue) as well as net [^{3}H]preS1 binding rates (shown in red), which are expressed as % of control at the y-axis. IC_{50} values were calculated by nonlinear regression analysis using the equation log(inhibitor) vs. response (GraphPad Prism). Data represent means ± SD of quadruplicate determinations of representative experiments.

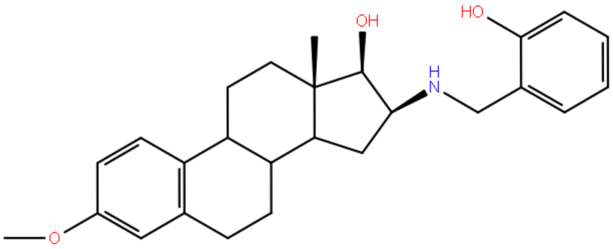

steroid 8c

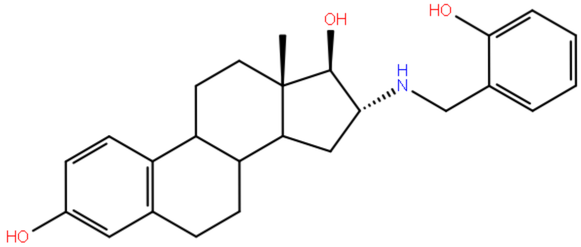

steroid 9c

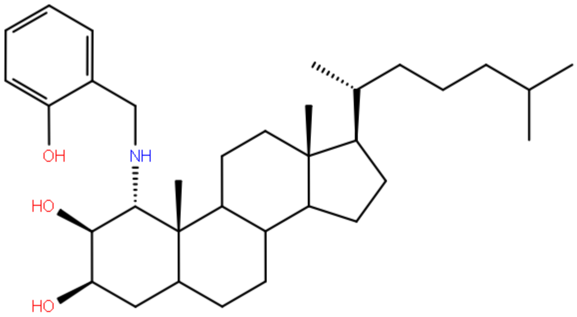

steroid 12c

Supplementary Figure S2. Structures of selected arylmethylamino steroids with highest potency of [^{3}H]preS1 peptide binding inhibition. All structures can be found in the publication by Krieg et al. (2017).

Supplementary Figure

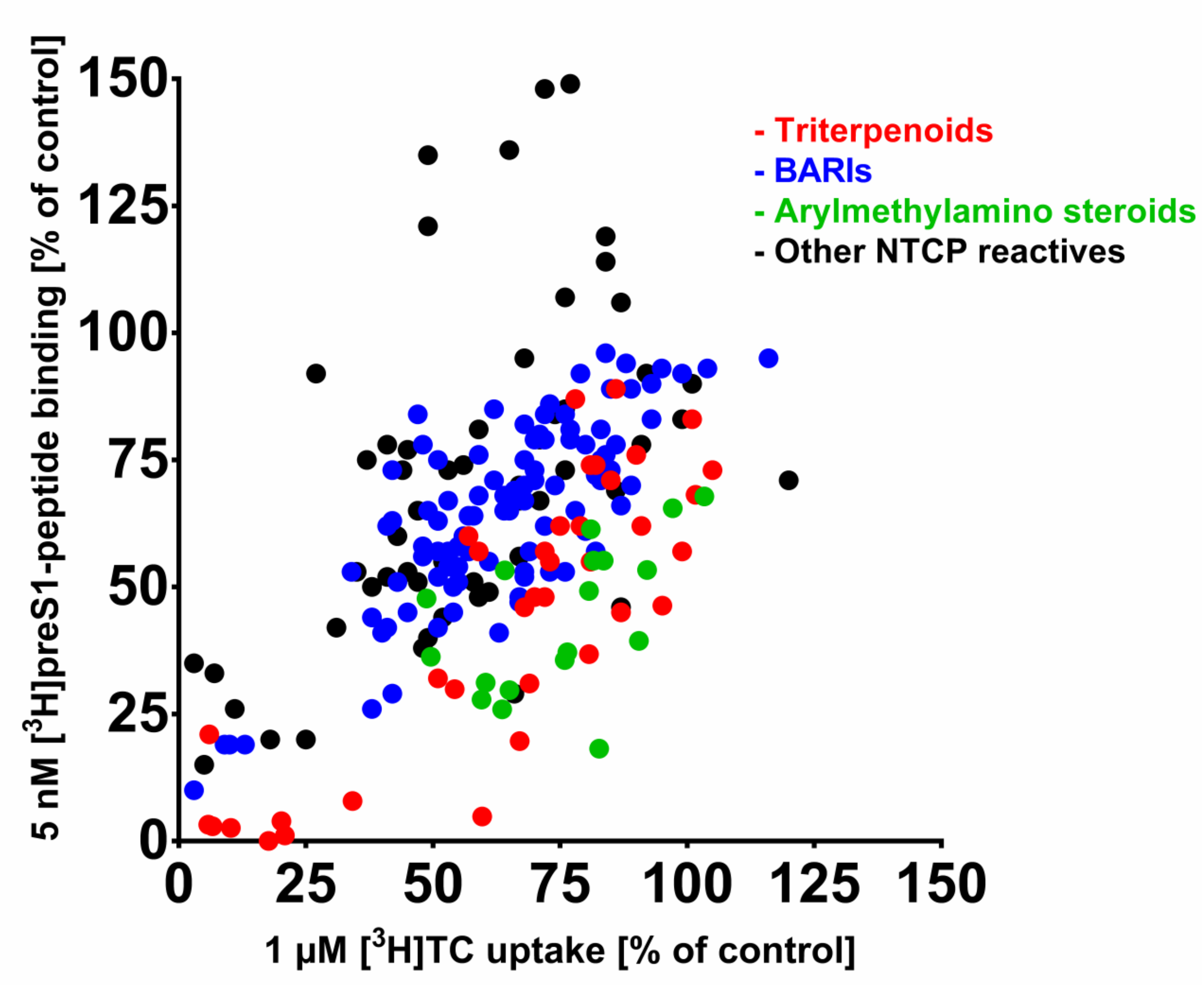

Supplementary Figure S3. Residual [^{3}H]TC transport activity via NTCP vs. residual [^{3}H]preS1-peptide binding to NTCP in the presence of inhibitors. All compounds were used at 100 µM inhibitor concentrations in transport assays with 1 µM [^{3}H]TC and binding assays with 5 nM [^{3}H]preS1-peptide, both in NTCP-HEK293 cells for 10 min at 37°C.

Reference

Krieg, R.; Jortzik, E.; Goetz, A.A.; Blandin, S.; Wittlin, S.; Elhabiri, M.; Rahbari, M.; Nuryyeva, S.; Voigt, K.; Dahse, H.M.; et al. Arylmethylamino steroids as antiparasitic agents. *Nat Commun* **2017**, *8*, 14478, doi:10.1038/ncomms14478.
